# Supplementary material for: Transcriptional read through interrupts boundary function in Drosophila
Source: bioRxiv. 2023 Feb 16:2023.02.16.528790. Preprint. [Version 1] doi: 10.1101/2023.02.16.528790 (PMC9949125; doi:10.1101/2023.02.16.528790)
Supplement: Supplement 1 [file NIHPP2023.02.16.528790v1-supplement-1.pdf]

bioRxiv preprint doi: https://doi.org/10.1101/2023.02.16.528790; this version posted February 16, 2023. The copyright holder for this preprint (which was not certified by peer review) is the author/funder, who has granted bioRxiv a license to display the preprint in perpetuity. It is made available under aCC-BY 4.0 International license.

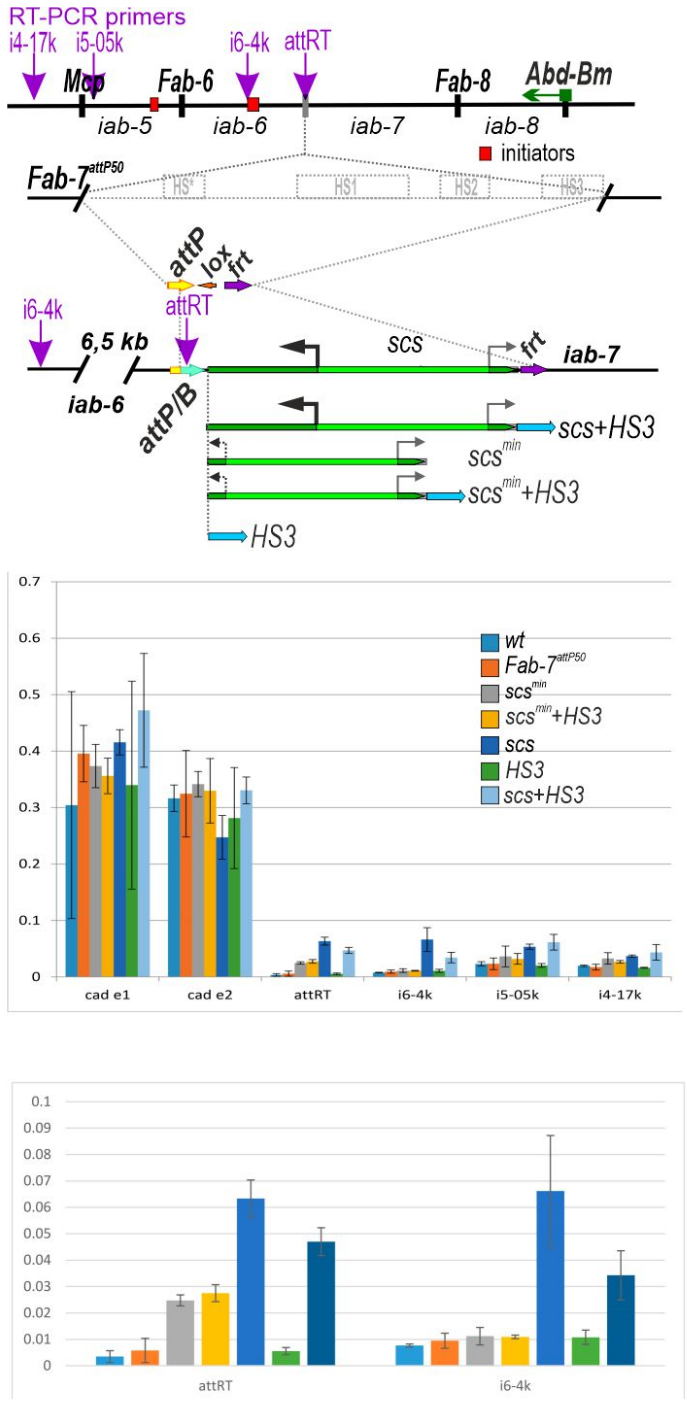

787

788 **S1 Fig.**

bioRxiv preprint doi: https://doi.org/10.1101/2023.02.16.528790; this version posted February 16, 2023. The copyright holder for this preprint (which was not certified by peer review) is the author/funder, who has granted bioRxiv a license to display the preprint in perpetuity. It is made available under aCC-BY 4.0 International license.

SI Table 1. Primers for generating fragments

**Fragments**	**Primers**	**5' - 3'**
**scs**	scs d	CGCTGCGAACTTCTCTTC
	scs r	CTGTATTCCTCAGTTATCGA
**scs^{min}**	scs m d	CGTCCGCATACGTCCG
	scs r	CTGTATTCCTCAGTTATCGA
**F7 HS3**	HS3_d	GTCGCAAGAACTTCACAACAG
	HS3_r	GCCATCATGGATGTGAAAGA
**PAS (sv40)**	sv40tr	GATACATTGATGAGTTTGG
	sv40td	GGATCTTTGTGAAGGAACCTTAC
**5'P**	1147	CATGATGAAATAACATAAGGTGGTC
	1152	GCTGCTGCTCTAAACGACG
**F7^{1+2+3}**	F7-1	GATTTCAAGCTGTGTGGCGGGGG
	F7-3	ATGTCGGCAATTCGGATTCCCGG
**F2 pHS2**	F2-47	TTTGTGAATCCGTACCC
	F2-48	TGAGCGAGTCCTTGAG
**F2**	F2D	GCTGAGGCGGCTGAGAAAG
	F2R	CAAGATACAATCAGCAAAGC

SI Table 2. The sequences of oligonucleotides used in real-time PCR

Vha_RT2d	TCATCTTCCACAACGCTTAC
Vha_RT2r	GGAGATCCTGTTCCTGAAATAC
cad_e1_RTd	CCTGTCTGTGTTGGTGTTAT
cad_e1_RTr	TCATTCACACACCAGCTTT
cad_e2_RTd	CGGCATTGGAGAAGACAA
cad_e2_RTr	GGCTGATGACGTTGGAAT
i4-17k_d	TTTCAATGGCGGACGTATC
i4-17k_r	CCGCACTTGACTCTTGTTAT
i5-05k_d	CGGCAATACTCAAGGTTTCT
i5-05k_r	CTTCGTTCCTCGCTTTATGT
i6-4k_d	CGACCTCCTTGTGTTGATTT
i6-4k_r	TCAGATGACACCTCCCTTT
attRT_dir	GTGGCGGTAGTTGATCCC
attRT_rev	GTCGAGAACCCGCTGAC
